# Bioactive Potential of Tocosh Supplemented with Selenium-Enriched Saccharomyces Cerevisiae Biomass

**DOI:** 10.3390/foods14183153

**Published:** 2025-09-10

**Authors:** Gilmar Peña-Rojas, Edgar Escriba-Gutierrez, Vidalina Andía-Ayme, L. Mateo Cordero-Clavijo, Marco A. Lazo-Vélez

**Affiliations:** 1 Laboratorio de Biología Celular y Molecular, Universidad Nacional de San Cristóbal de Huamanga, Portal Independencia 57, Ayacucho 05003, Peru; 2 Laboratorio de Microbiología de Alimentos, Universidad Nacional de San Cristóbal de Huamanga, Portal Independencia 57, Ayacucho 05003, Peru; edgar.escriba.03@unsch.edu.pe (E.E.-G.); vidalina.andia@unsch.edu.pe (V.A.-A.); 3 Grupo de Investigación NutriOmics, Universidad del Azuay, Av. 24 de Mayo 7-77 y Hernán Malo, Cuenca 010150, Ecuadormalv@uazuay.edu.ec (M.A.L.-V.)

**Keywords:** selenium, antioxidant, anti-inflammatory, fermented potato

## Abstract

Tocosh is a traditional Andean food made from fermented potatoes with remarkable nutritional and antibacterial properties. Selenium (Se) is an essential micronutrient associated with antioxidant and anti-inflammatory activities. This study analyses the in vitro glycemic, antioxidant, anti-inflammatory and cytotoxic potential of tocosh supplemented with Se-enriched *Saccharomyces cerevisiae* biomass produced using various concentrations of sodium selenite (0, 5, 7 and 9 mg/L) over 72 h. For each treatment, the amount of selenium accumulated and the yeast’s ability to incorporate selenium were determined. Se-enriched tocosh from yeast biomass obtained at a concentration of 5 mg/L at 4, 16 and 28 h was used to evaluate the aforementioned bioactive properties. These concentrations promoted greater cell growth and biomass recovery without compromising selenium incorporation. No significant differences were observed in terms of glycemic index and antioxidant capacity in Se-enriched tocosh, but inhibition of NO production was observed in RAW 264.7 cells treated with tocosh enriched with selenium-enriched yeast biomass obtained after 28 h, with an IC_50_ of 28 mg/mL. Overall, Se-supplemented tocosh exhibited anti-inflammatory properties, highlighting the potential of integrating Se biofortification into traditional Andean foods as a novel approach to address nutrient deficiencies and modulate immune responses. These findings suggest that Se-enriched tocosh could be used as a functional food ingredient or nutraceutical in dietary interventions aimed at reducing chronic inflammatory disorders.

## 1. Introduction

Tocosh is a traditional and functional Andean food produced by fermenting potatoes (*Solanum tuberosum*) or other agricultural products. It is prepared by submerging the potatoes in cold spring water for weeks to allow the native microbiota to acidify and biotransform the matrix. Once fermentation is complete, the tubers are washed, dried in the sun and ground into flour used in porridges and beverages. This process reduces the pH, remodels starch and enhances the quantity and profile of organic acids, phenolic compounds and small peptides. These bioactive components are responsible for its therapeutic potential and act at various physiological levels [1,2].

Fermentation promotes the growth of lactic acid bacteria (LAB), which acts as probiotics, and encourages the formation of resistant starch, which functions as a prebiotic. This natural symbiotic process is essential for gastrointestinal health, enhancing starch digestibility and nutrient bio-accessibility [3]. Additionally, tocosh contains phenolic compounds with antioxidant properties that combat oxidative stress, which is linked to its anti-inflammatory properties [1,2]. Furthermore, hydroalcoholic extracts of tocosh have been shown to exhibit clear antibacterial activity against *Streptococcus mutans* [3]. Toxicological evaluations in rodents report no adverse effects at high doses (1000 mg/kg for 28 days), indicating a favorable safety margin for development [2]. Recent research also re-evaluates tocosh as a refined indigenous biotechnology with potential nutraceutical and probiotic value [4]. Together, the synergy of LAB-driven metabolite production, antimicrobial effects and starch remodeling establishes tocosh as a promising ingredient for next-generation functional foods, although more clinical research is needed to validate its effects in humans.

In addition, Se is an essential micronutrient for human and animal health, recognized for its role as a component of selenoproteins, whose functions include antioxidant defense, immune regulation, and cellular metabolism [5]. Enzymes such as glutathione peroxidases (GPx) neutralize reactive oxygen species (ROS), protecting cells from oxidative damage and contributing to the modulation of inflammatory responses. Selenium deficiency has been linked to an increased risk of cardiovascular disease, neurological disorders, and immune dysfunction. However, although Se is found in nature in inorganic forms, these have low bioavailability and some toxic potential; therefore, its conversion to organic forms such as selenomethionine is key to improving its absorption and biological efficacy [6,7]. Therefore, a promising biotechnological strategy for this purpose involves the selenization of yeasts such as *S. cerevisiae* [8,9]. These yeasts can convert up to 90% of the assimilable sodium selenite in the culture medium into stable, safe organic compounds (L-SeM), leaving only trace amounts of inorganic Se. In *S. cerevisiae*, Se is incorporated through the activation of selenate (SeO_4_^2−^) by ATP sulfurylase to form adenosine-5′-phosphoselenate (APSe), which is subsequently reduced to selenite (SeO_3_^2−^) and then converted to reduced selenium (H_2_Se) through non-enzymatic reactions involving glutathione (GSH). Intermediate metabolites, including GSSeO_2_^3−^ and GSSe, are produced at this stage. Selenocysteine (SeC) is synthesized from H_2_Se and can be incorporated into selenoproteins or transformed into selenomethionine (SeM). However, this capacity is limited by the inhibitory effect of high Se concentrations in the culture medium. Additionally, homoserine may serve as an alternative SeM precursor. In contrast, higher organisms such as humans lack the biosynthetic capacity for methionine, meaning it must be obtained through diet [8,10].

As part of the development of innovative strategies to improve the nutritional value of traditional foods, this research explored the potential synergy between tocosh and selenium-enriched biomass obtained from a commercial strain of *Saccharomyces cerevisiae*. Different concentrations of sodium selenite (5, 7 and 9 mg/L) and fermentation times were evaluated to determine their impact on yeast cell growth, selenium-enriched biomass production and intracellular selenium content. The aim was to identify the optimal conditions for producing a safe and functional yeast. The resulting biomass, which is rich in organic selenium compounds with high bioavailability, has been suggested as a biofortifying agent for tocosh to enrich its functional profile. The biological value of the final product will subsequently be evaluated in vitro using parameters such as glycemic index, antioxidant capacity, cytotoxicity and anti-inflammatory activity.

## 2. Materials and Methods

### 2.1. Selenium-Enriched Yeast Biomass

Eleven milligrams of dry S. cerevisiae (Instant Success yeast strain, Lesaffre, Peru; lot L22B345), with an initial count of ≥1 × 10^9^ colony-forming units (CFU)/g, was cultured in a 250 mL Erlenmeyer flask containing 50 mL of sterile YEPD (yeast extract–peptone–dextrose), which was prepared following the standardized protocol [11]. Briefly, the YEPD medium contained 10.0 g/L of yeast extract, 20.0 g/L of peptone, and 20.0 g/L of dextrose dissolved in sterile distilled water to give a final volume of one liter. The nutritional composition of the YEPD medium was determined to be 10.44 ± 0.20 g/L of carbon, 3.27 ± 0.15 g/L of nitrogen, 0.22 ± 0.01 g/L of phosphorus, and 21.50 ± 0.10 mg/L of sulfur.

The flask containing yeast plus YEFP was placed in a boiler at 36 ± 1 °C for 20 min to activate the yeast. Additional YEPD solution was then added to adjust the flask’s volume, after which the flask was shaken at 120 rpm and 30 °C for 24 h. The inoculum from the previous step, with initial cell density values of ≥2.2 × 10^5^ CFU/mL, was added to a 50 mL Falcon tube containing 45 mL of sterile YEPD liquid medium in a laminar flow chamber. A total of 64 tubes were used for colony counting and 9 for biomass determination and total intracellular Se quantification. After inoculation, the tubes were placed on a shaker at 30 °C and 120 rpm for five hours of growth. Then, volumes of sodium selenite solution were added. Stirring was continued under the same conditions in all experimental tubes except the first sixteen to obtain concentrations of 0, 5, 7 and 9 mg/L. Three replicates were performed for each experiment. The sodium selenite solution was prepared under sterile conditions at a concentration of 1000 mg Se(IV)/L, as described in reference [9]. Finally, after 72 h, the yeast cultures were placed in 50 mL Falcon tubes. The yeast biomass was then separated from the culture medium by centrifugation at 4000 rpm for five minutes [12,13]. The recovered yeast biomass was then stored at 2 °C for total Se determination.

The growth of *S. cerevisiae* was monitored by counting the number of CFU/mL using the Sabouraud agar plating technique. According to [11], samples were collected at time zero (after inoculation) and at regular intervals of every 4 h for the first 48 h, and then every 8 h until 72 h of culture were completed. The selection of 4, 16, and 28 h was made due to their correspondence with the start of the exponential and stationary phases and the point at which the decline phase of yeast growth is determined, respectively. Thereafter, one mL of the 1:10 dilution corresponding to each treatment (0, 5, 7, and 9 mg/L of sodium selenite) was used, and the plates were incubated at 30 °C for 24 h, and the number of colonies was quantified using a manual colony counter. This procedure generated accurate growth curves that evaluated the effect of each selenite concentration on cell proliferation kinetics.

### 2.2. ICP-OES Selenium Quantification

The total Se-enriched yeast biomass was determined through open-system acid digestion, followed by analysis using inductively coupled plasma optical emission spectrometry (ICP-OES; Optima 8000, PerkinElmer^®^, Waltham, MA, USA) at a wavelength of 196.0 nm. Aliquots of 0.5 g of the sample were subjected to a three-stage stepwise digestion procedure involving nitric acid and hydrogen peroxide. The total volumes of the acids were 15 and 10 mL, respectively. The stages were: (1) pre-digestion at room temperature for 24 h, (2) primary digestion at 70 °C for 10 min, and (3) secondary digestion with successive additions of oxidizing agents at 70 °C for 60 min. The resulting digests were filtered with 0.45-μm membrane filters, brought to a final volume of 25 mL with 2% *v*/*v* HNO_3_, and analyzed in triplicate.

Selenium concentrations were determined by interpolation from a calibration curve and expressed as mg/kg of dry weight, with relative standard deviations (RSD) below 5% among replicates. Aqueous Se standards (0.00, 0.5, 1.0, 2.0, and 5.0 mg/L) were prepared by serial dilution of a certified 1000 mg/L stock solution to construct the calibration curve. An acidic matrix identical to that used in the sample digestion (HNO_3_:H_2_O_2_, 3:1 *v*/*v*) was employed for this dilution. The calibration blank (n = 2) exhibited corrected signal intensities of −64.3 ± 3.66 (RSD = 5.48%), confirming instrumental stability. The ICP-OES method was validated using recovery experiments with SELM-1 certified reference material (CRM) (selenium-enriched yeast, National Research Council of Canada). Aliquots of the CRM were subjected to the same acid digestion protocol as the unknown samples. A mean recovery value of 98.2% was obtained, demonstrating the high precision of the method and confirming the efficiency of the digestion process in releasing selenium from the biological matrix. Measurements were performed in duplicate with intermediate rinsing using the same acidic matrix. Corrected emission intensities were recorded using WinLab32 software. The linear regression model yielded an R^2^ greater than 0.995, demonstrating the method’s linearity within the evaluated concentration range.

### 2.3. Selenium-Enriched Tocosh

To prepare the mixtures, 9.5 g of commercial tocosh flour (Ayacucho, Peru) were combined with 0.5 g of lyophilized selenium-enriched yeast biomass. This corresponded to a selenium concentration of 5 mg/L, which was obtained from cultures harvested at 4, 16, and 28 h. The mixture was then homogenized mechanically under aseptic conditions to ensure uniform distribution. The resulting samples were stored in aluminum-laminated bags at 2 °C in the dark until further analysis.

### 2.4. In Vitro Glycemic Index

The method described by [14] was used to determine the estimated glycemic index in vitro. First, 0.5 g of each selenium-enriched tocosh sample (control, 4 h, 16 h, and 28 h) was weighed into 15 mL Corning tubes. Then, 10 mL of sodium acetate buffer solution (pH 5.2) was added. The suspensions were then incubated in a shaking incubator at 37 °C and 120 rpm (JSR, Crawley, England). Then, a solution of pancreatic α-amylase (75 U/mL) and amyloglucosidase (50 U/mL) was prepared and 100 μL of the solution was added to each tube. The samples were then incubated at 37 °C and 120 rpm. Aliquots were taken at 10, 20, 30, 60, 90, and 120 min for glucose quantification using the Glucose reagent kit (Megazyme, Ireland). Then, equation described by [11] was used to extrapolate the first-order kinetic equation:

C = C (1 − e^−kt^) (1)

where C represents the percentage of starch hydrolyzed at time t (min); C represents the equilibrium percentage of starch hydrolyzed after 120 min; and k represents the kinetic constant.

The parameters C and k were estimated for each product based on data obtained from in vitro digestion. The hydrolysis index (HI) was derived by dividing the area under the curve (AUC) of each sample’s hydrolysis by the AUC of the global reference food, the white bread. The estimated glycemic index (eGI) was calculated using the following equation:

eGI = 39.71 + 0.54HI(2)

### 2.5. Simulated In Vitro Gastrointestinal Digestion

The control and Se-enriched tocosh samples were subjected to in vitro simulated gastrointestinal digestion (SGID) to evaluate their bioactive potential. Digestive suspensions with their corresponding enzymes were prepared to simulate the mouth, stomach, and intestines according to the procedure reported by [15]. All SGID steps were performed in an incubator (JSR, Crawley, England) at 37 °C with stirring at 120 rpm. In brief, 2 g of each ground tocosh sample was weighed into 50 mL Corning centrifuge tubes and mixed with 2 mL of mouth suspension containing α-amylase (75 U/mL). The suspension was then incubated for 10 min at 37 °C while stirring at 120 rpm. Then, 10 mL of the gastric suspension (pH 3.0 ± 0.2) containing porcine pepsin (2000 U/mL) was added, and the tubes were further incubated for two hours. For intestinal digestion, 15 mL of intestinal suspension (pH 7.0 ± 0.2) containing porcine pancreatin (100 U/mL), lipase (15 U/mL), and bovine bile salts (10 mM) were added to each tube. The tubes were then incubated for two more hours. To stop the activity of the digestive enzymes, the tubes were placed in an ice bath. After digestion, the tubes were centrifuged at 9000 rpm for 15 min at 4 °C. The resulting supernatant was recovered and ultra-frozen at −80 °C for 24 h. Finally, the digested samples were lyophilized for 96 h at −80 °C and 100 Pa.

### 2.6. DPPH Radical Scavenging Activity

The antioxidant capacity was evaluated using the DPPH radical scavenging activity method as described by [16], with minor modifications. Briefly, 48 mg of each gastrointestinal-digested sample were extracted with 1 mL of 80% methanol (*m*/*v*) by agitation for 2 h at 40 rpm using a circular roller (Fisherbrand, Barcelona, Spain). Following extraction, the suspensions were centrifuged at 10,000 rpm for 10 min, and the resulting supernatants were collected for antioxidant capacity assessment. For the assay, 50 µL of each methanolic extract were mixed with 1.95 mL of a 60 µM DPPH solution prepared in methanol. The mixtures were incubated in the dark at room temperature for 30 min, and absorbance was subsequently measured at 517 nm using a UV–Vis spectrophotometer. The antioxidant capacity was expressed as equivalents of mM Trolox per g of SGID tocosh.

### 2.7. In Vitro Nitric Oxide Inhibition

The anti-inflammatory potential of Se-enriched tocosh samples was evaluated in vitro on murine macrophage cells (Raw 264.7) as previously described by [17]. Briefly, the Raw 264.7 cells were cultured in DMEM (Gibco^TM^, Waltham, MA, USA) supplemented with 5% FBS and 1% of the commercial streptomycin-penicillin mixture (Gibco^TM^, Waltham, MA, USA) and incubated at 37 °C in a 5% CO_2_ incubator. Cells were seeded in 96-well plates (5 × 10^5^ cells/mL) and allowed to adhere for 24 h. The next day, 50 μL of SGID digested tocosh samples (100 mg/mL DMEM) were added to the cells. After 4 h, half of the wells in the 96-well plate were stimulated with lipopolysaccharides (LPS) from *Salmonella* enterica serotype typhimurium (L7261, Sigma-Alrich, St. Louis, MO, USA) at 10 μg/mL (final concentration), while the other half were used as controls for each sample. The cells were further incubated with LPS for 24 h at 37 °C and 5% CO_2_. The nitric oxide (NO) production was indirectly measured by means of nitrite determination, using the Griess Reagent System (Invitrogen, Carlsbad, CA, USA). Absorbance measures were performed at 550 nm according to the manufacturer’s instructions using a microplate reader (Epoch, BioTek Instruments, Winooski, VT, USA). The results were obtained in triplicate and expressed as a percentage of NO inhibition.

### 2.8. In Vitro Cytotoxicity Assay

The cytotoxic effects of Se-enriched tocosh samples were evaluated on human colorectal adenocarcinoma cells (Caco-2) according to [18]. The Caco-2 cells were cultured in DMEM (Gibco™, Waltham, MA, USA) supplemented with 5% FBS and 1% commercial streptomycin-penicillin (Gibco™, Waltham, MA, USA) mixture and incubated at 37 °C in a 5% CO_2_ incubator. The cells were seeded at a density of 5 × 10^4^ cells/mL in 96-well plates and allowed to adhere for 24 h. Then, 100 µL of digested Se-enriched tocosh samples ranging from 3 to 200 mg of SGID tocosh/mL were added to the cells and incubated for an additional 48 h. Finally, cell viability was measured using the CellTiter 96 Aqueous One Solution Kit (Promega Corporation, Madison, WI, USA). Cell viability was calculated by dividing the absorbance of the treated cells by the absorbance of the control (untreated) cells. This ratio was then expressed as a percentage. The study was performed in triplicate.

### 2.9. Statistical Analysis

Data analysis was performed with Minitab^®^ Statistical Software (version 21). A one-way ANOVA was applied to compare the effects of sodium selenite concentrations (0, 5, 7 and 9 mg/L) on cell growth, biomass and intracellular Se. Depending on the distribution of the data, Tukey’s or Dunnett’s post hoc tests were applied, considering a significance level of *p* < 0.05.

## 3. Results and Discussion

### 3.1. Obtaining Selenized Biomass

**Figure 1:**
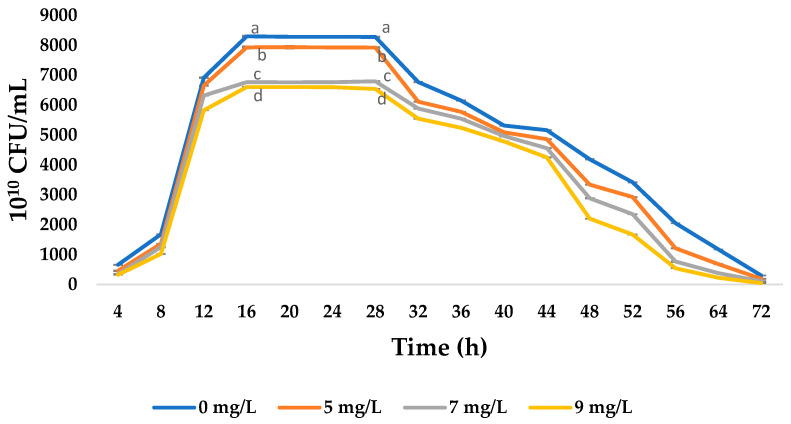
Effect of sodium selenite on the growth of *Saccharomyces cerevisiae* biomass. Values are expressed as mean ± standard deviation (n = 3). At each time point, means followed by different lowercase letters differ significantly (*p* < 0.05, Tukey’s HSD test).

*Saccharomyces cerevisiae* cultures supplemented with different concentrations of sodium selenite (0, 5, 7 or 9 mg/L) exhibited significant behaviors in terms of cell growth (CFU/mL), biomass production, and intracellular Se accumulation (*p* < 0.05). As shown in Figure 1, the growth curve followed typical batch culture kinetics, with an exponential phase occurring between 4 and 16 h, a stationary phase from 16 to 28 h and a decline phase thereafter. The highest cell count was obtained at a concentration of 5 mg/L, reaching a maximum value of 8424 CFU/mL after 16 h. In contrast, the 7 and 9 mg/L concentrations clearly inhibited growth, which revealed statistically significant differences (*p* < 0.05).

Therefore, a concentration of 5 mg/L of sodium selenite produced the most effective selenized biomass after 16 h, whereas higher concentrations decreased cell viability (see Figure 1). Previous studies have reported similar efficiencies in converting selenite into organic forms for biomass production at concentrations of 5–8 mg/L. However, yeast growth was inhibited at concentrations of 10–12 mg Se/L [8,12,19,20]. In line with these findings, ref. [21] reported that Se incorporation into *S. cerevisiae* cells caused severe toxicity at 20 mg/L, while [8] suggested that, under low pH conditions and above this concentration, elemental Se can be generated, imparting a red color and metallic taste to the biomass. A concentration of 8 mg/L promoted high cell viability and an intracellular Se content of 738 mg/kg (dry basis) in the S. cerevisiae LALVIN RC-212 strain; however, growth inhibition was observed between 8 and 10 mg/L [22]. While time and concentration are both key factors in Se accumulation in yeast, variability in Se concentrations can also be attributed to factors such as the composition of the culture medium, the stage at which selenite is added and inherent phenotypic variability between commercial, bakery and laboratory strains [23]. Additionally, ref. [13] reported that Se tolerance depends heavily on the strain used due to variations in anion transport systems, antioxidant enzyme production and detoxification mechanisms. For example, *Candida utilis* exhibited a higher bioaccumulation capacity than *S. cerevisiae* under comparable conditions, and native or genetically modified strains may also exhibit greater biotransformation capacity [24]. Overall, these findings imply that, while *S. cerevisiae* is an effective model organism for producing selenized yeast, its response can be influenced by genotypic and phenotypic factors.

**Figure 2:**
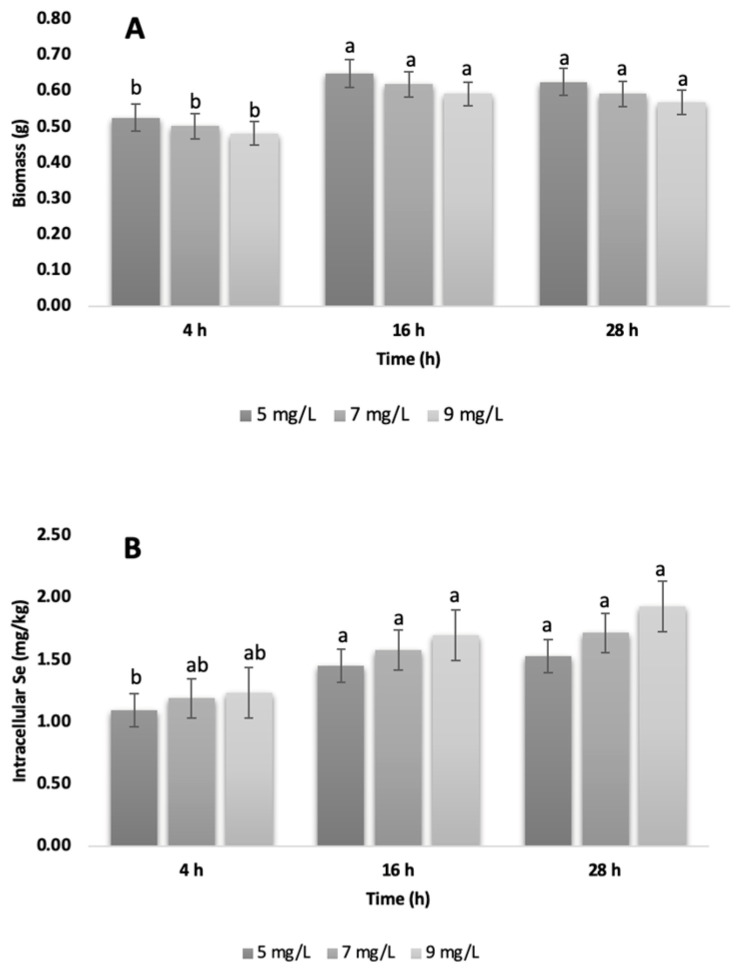
(**A**): Biomass generation (g); and (**B**): Quantification of total intracellular Selenium (mg/kg) in the biomass of *Saccharomyces cerevisiae*. Values are expressed as mean ± standard deviation (n = 3). Within each graph, bars with different lowercase letters are significantly different according to Tukey’s HSD test (*p* < 0.05). For comparison, the basal Se level reported for *Saccharomyces cerevisiae* without Se supplementation (~0.862 mg/kg dw) [26] can be considered as a reference value.

Regarding biomass production (Figure 2A), a pattern consistent with the cell growth results was observed. Although quantitative differences were evident, ANOVA did not show statistical significance (*p* > 0.05), which can be attributed to high intra-group variability. In more detail, variation in the cell cycle in batch cultures generates subpopulations with different metabolic capacities for tolerating and accumulating selenium. Consequently, selenite induces oxidative stress unevenly, with some cells activating detoxification mechanisms (e.g., increased glutathione production) and others suffering irreversible damage. The potential flocculation of this commercial strain under stress, and/or the volatilization of selenized compounds (e.g., dimethylselenide), could create inhomogeneities in the culture and cause loss of different analytes [25]. Nevertheless, the 5 mg/L concentration promoted the highest biomass production during the stationary phase (16 h), suggesting that this concentration and time optimize selenization without compromising cell viability. In the present work, the maximum biomass was obtained at 16 h (early stationary phase), a stage in which the cell has completed its exponential division and channels resources towards secondary metabolic processes, such as selenoamino acid synthesis and intracellular storage of Se. This observation is in agreement with that described by [12], who found that growth phase and carbon source directly influence selenization efficiency.

### 3.2. ICP-OES Selenium Quantification

The results for intracellular Se content are presented in Figure 2B. No statistically significant differences between treatments were observed (*p* > 0.05). However, a progressive increase in Se levels in biomass was detected as the selenite concentration increased. This suggests that, although cells accumulate Se efficiently, there is a tolerance threshold beyond which cell viability is compromised. Therefore, producing functional selenized biomass requires an appropriate selenite concentration (5–8 mg/L is tolerable for many strains), optimal timing of harvest (early stationary phase) and the correct strain with the physiological capacity to resist oxidative stress and transform Se [26,27]). High concentrations generate reactive oxygen species (ROS), inducing oxidative stress, membrane disruption, and cell death [28]. This phenomenon may explain the observed decrease in CFU/mL count and biomass after 28 h, when the cells enter the decline phase. Authors as [29] emphasize that optimizing the enrichment process is essential to prevent toxicity and ensure efficient conversion to organic forms. Therefore, strict control of these factors is crucial to maximizing selenoprotein production while avoiding toxic effects. However, this study focused on the robust quantification of total intracellular selenium as a first step in establishing optimal growth and accumulation parameters for this specific commercial strain. The high reported accumulation levels, together with preserved cell viability in the 5 mg/L treatment, strongly suggest effective biotransformation of inorganic selenite (SeIV) into less reactive organic forms. This inference is based on *S. cerevisiae’s* well-established metabolic pathway, in which selenite is reduced by glutathione to hydrogen selenide (H_2_Se), a precursor to selenoamino acids, such as selenomethionine (SeMet) and selenocysteine (SeCys) [25]. For future work aimed at producing selenized biomass, we recommend targeted Se speciation, e.g., high-performance liquid chromatography–inductively coupled plasma–mass spectrometry (HPLC–ICP-MS) analysis of SeMet, SeCys, and residual Se (IV), to confirm biotransformation, guide process optimization, and ensure bioavailability and safety.

Moreover, recent evidence suggests that polyphenols and fermentation metabolites can alter the behavior of Se in fermented foods such as tocosh. Catechins and related phenolic compounds reduce Se (IV) and alter its speciation, turning it into elemental selenium or selenium nanoparticles (SeNPs) capped with polyphenols. These changes can affect dissolution, intestinal transport and redox behavior [30]. Such SeNPs often have a phenolic structure that stabilizes the particles and enhances cellular uptake via endocytic routes, compared with ionic/organic Se. In parallel, LAB-driven fermentation converts selenite into selenomethionine and selenocysteine, further remodeling the available Se pool [31]. The effects of processing matter as well: sourdough fermentation increases the bioavailability of organic Se, and recent in vitro digestion/fermentation studies show that Se bioavailability is matrix-dependent [32]. Together, these data highlight the need for further targeted studies on tocosh to determine whether its native phenolic compounds and fermentation products influence Se speciation, bioaccessibility and epithelial uptake.

### 3.3. In Vitro Glycemic Index

**Figure 3:**
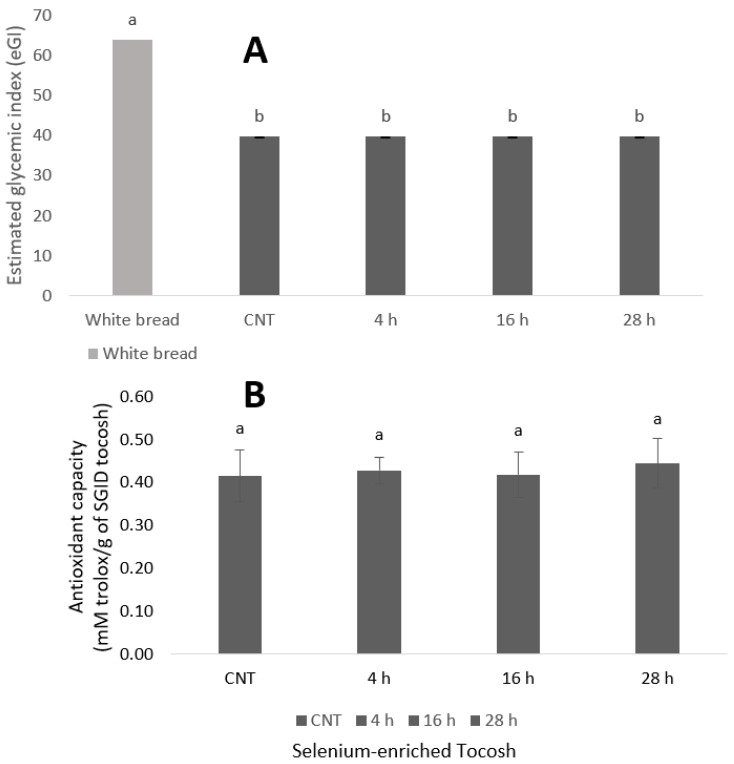
(**A**): Estimated in vitro glycemic index, and (**B**): Antioxidant activity of selenium-enriched tocosh. Values are expressed as means ± SD of at least three independent replicates (n = 3). Within each graph, bars with different lowercase letters are significantly different according to Tukey’s HSD test (*p* < 0.05).

The estimated glycemic index (eGI) of all tocosh samples (Figure 3A) was significantly lower than that of white bread (eGI ≈ 65–70) (*p* < 0.05), with no significant differences observed between the Se-enriched tocosh treatments and the non-enriched control (CNT). Consistent with prior reports indicating no conclusive effect of Se biofortification on glycemic response [23], this outcome is mechanistically plausible because GI is governed primarily by starch architecture and matrix effects (resistant starch formation, amylose/amylopectin ratio, retrogradation, and fiber–phenolic entrapment), whereas Se enrichment occurs mostly within yeast proteins (SeMet/SeCys) at microgram-per-gram levels that neither alter starch gelatinization/viscosity nor meaningfully inhibit α-amylase/α-glucosidase. Thus, the carbohydrate fraction and fermentation-derived microstructure dominate hydrolysis kinetics, overshadowing any Se-specific influence at the tested doses. Still when compared to other tuber-based matrices, such as taro-eGI ≈ 63 [33] and roasted cocoyam-eGI ≈ 68 [34], tocosh demonstrated a significantly lower glycemic response. Notably, recent studies have emphasized that low-glycemic index diets can enhance insulin sensitivity, reduce systemic inflammation and blood pressure, and lower the risk of developing metabolic diseases [35]. These results support the potential application of tocosh as a functional ingredient, particularly in formulations targeted at individuals with, or at risk of, type 2 diabetes or metabolic syndrome, or the development of functional foods aimed at glycemic control.

### 3.4. Antioxidant Activity

The antioxidant capacity of tocosh, as measured by the DPPH radical scavenging assay (Figure 3B), ranged from 0.41 to 0.45 mM Trolox/g of SGID tocosh, with no statistically significant differences (*p* > 0.05) between the control sample (CNT) and the Se-enriched treatments (4 h, 16 h, and 28 h). These findings indicate that the Se biofortification strategy implemented in this study did not enhance nor impair the radical scavenging activity of tocosh. The antioxidant values across treatments may be attributed to the intrinsic phytochemical composition of the fermented tocosh matrix. Previous studies on tocosh, traditionally fermented potato, have identified phenolic compounds, flavonoids, and steroidal or triterpenoid secondary metabolites as key contributors to its antioxidant potential, both in vitro and in chemical assays [36]. Comparable observations have been reported by [37], who evaluated Se biofortification in microgreens (kale, kohlrabi, and wheat) and found that total antioxidant activity remained unchanged. The authors suggested that the absence of bioactivity shifts could be due to differences in species-specific metabolism, developmental stage, or the nature and dose of Se treatments, factors that may also be influencing the present findings. It is possible that higher Se concentrations or alternative delivery strategies would be required to observe any measurable impact on antioxidant activity in tocosh. Nevertheless, the overall antioxidant capacity consistently observed across all treatments supports the notion that the health-promoting properties of tocosh may be primarily driven by its native bioactive compounds. Nevertheless, the DPPH assay is convenient but probes only one stable radical in organic media and mainly single-electron-transfer reactions. Outcomes are sensitive to solvent/pH, reaction time/kinetics, and matrix color/turbidity. Future work should use a multi-assay panel to gain insight into the mechanisms. This panel should include ABTS and FRAP/CUPRAC (electron transfer), ORAC (hydrogen-atom transfer/chain-breaking), Fe^2+^-chelating capacity, and post-digestion, cell-based ROS assays (e.g., Caco-2 or RAW 264.7).

### 3.5. Anti-Inflammatory Effects of Se-Enriched Tocosh

**Figure 4:**
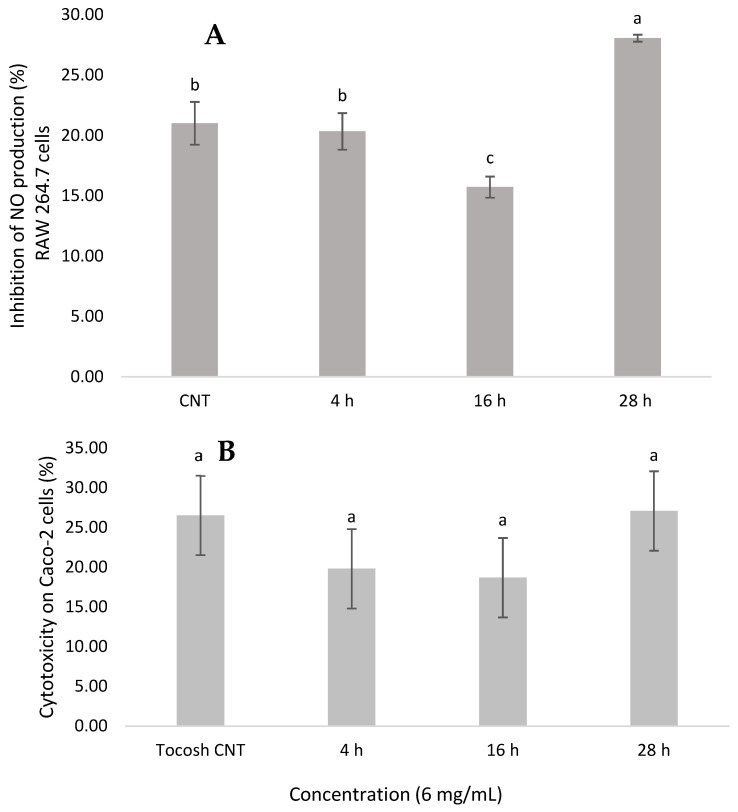
(**A**): The nitric oxide inhibition (%) of selenium-enriched tocosh in murine macrophage cells (RAW 264.7). (**B**): Cytotoxic effects (%) of the selenium-enriched tocosh on human colorectal adenocarcinoma (Caco-2). Values are expressed as mean ± standard deviation (n = 3). Within each graph, bars with different lowercase letters are significantly different according to Tukey’s HSD test (*p* < 0.05).

The results depicted in Figure 4A demonstrate that Se-enriched tocosh exhibited a significant inhibitory effect on NO inhibition in Raw 264.7 murine macrophage cells, indicating potential anti-inflammatory activity. Among the treatments, the 28 h Se-enriched tocosh showed the highest NO inhibition (~28%), which was significantly higher (*p* < 0.05) than all other treatments, including the CNT and the 4 h and 16 h samples. The 16 h treatment exhibited the lowest inhibitory activity (~16%). Moreover, the 4 h treatment did not differ significantly from the untreated control, indicating that short fermentation times may not be sufficient to enhance the anti-inflammatory potential of tocosh. These differences could be attributed to the combined effects of Se bioavailability and the dynamic profile of fermentation-derived secondary metabolites, such as phenolic compounds and peptides, which are known modulators of inflammatory pathways [38]. Previous studies have highlighted the role of Se in modulating immune responses through the regulation of key inflammatory mediators such as TNF-α, IL 6 and IL 1β, and COX-2 production [39]. The inhibitory effects have been positively correlated with Se content. The greater anti-inflammatory activity observed in the 28 h treatment could be attributed to the increased accumulation and biotransformation of selenium into bioactive organic forms. During the late stationary phase, yeast cells increase the expression of detoxifying enzymes, such as glutathione reductase and metallothioneins, in response to nutritional and oxidative stress. This favors the reduction of Se (IV) to Se (II) and its subsequent incorporation into SeMet and MeSeCys [24]. These organic compounds, particularly MeSeCys, are methylselenol precursors. Methylselenol is an effector metabolite with potent anti-inflammatory activity due to its ability to inhibit the NF-κB pathway and suppress inducible nitric oxide synthase (iNOS) [38]. Thus, the longer exposure time (28 h) allows for greater metabolic conversion toward these bioactive forms, thereby enhancing the inhibitory effect on NO production in macrophages.

### 3.6. Cytotoxicity

The cytotoxic effects and IC_50_ of Se-enriched tocosh samples are presented in Table 1. The results showed that among all the tested concentrations (3 mg/mL up to 200 mg/mL), all treatments resulted in 17–27% toxicity for Caco-2 cells growth. These results suggest a safety profile for all fermentation times tested. These observations can be confirmed by the IC_50_ values, with higher values indicating lower cytotoxicity. However, the 28 h sample presented the lower IC_50_ (22.8 mg/mL), suggesting a relatively increased cytotoxicity in human adenocarcinoma cells at prolonged fermentation times. These differences may be associated with the evolution of fermentation-derived metabolites and the accumulation of Se species, which can shift from beneficial to cytotoxic depending on their concentration and chemical form [31].

The lower IC_50_ of the 28 h treatment, coupled with its higher NO inhibition, supports that longer fermentation times enriches redox-active organoselenium species such as SeMet/MeSeCys, which can convert to methylselenol. These species can (i) attenuate NF-κB signaling, (ii) suppress pro-inflammatory pathways, and (iii) act synergistically with phenolic compounds and peptides to amplify anti-inflammatory effects [39]. However, the same redox potency that heightens signaling inhibition can narrow the viability window (lower IC_50_) via GSH depletion or mitochondrial stress at higher doses [40]. This results in greater anti-inflammatory activity but also slightly increased cytotoxicity. Therefore, a practical conclusion is that a selective dose and fermentation time window of 16–28 h maximize anti-inflammatory efficacy while preserving cell viability. Overall, the data support the biocompatibility of Se-enriched tocosh, especially at moderate fermentation times (4–16 h), making it a promising candidate for functional food or nutraceutical applications with low intestinal toxicity risk. Nevertheless, additional studies are needed to determine whether Se-enriched tocosh exhibits selective toxicity toward transformed or malignant cells, which could highlight its potential for targeted applications.

## 4. Conclusions

The present study shows that supplementation of tocosh with Se-enriched yeast (*S. cerevisiae*) biomass, particularly when produced with 5 mg/L sodium selenite, results in improved yeast growth and biomass recovery without reducing Se incorporation efficiency. While no significant changes were detected in the glycaemic index or antioxidant capacity of Se-enriched tocosh, a clear anti-inflammatory effect was observed, evidenced by the inhibition of nitric oxide production in RAW 264.7 cells treated with biomass obtained at 28 h (IC_50_ = 28 mg/mL). These findings confirm that yeast-based Se enrichment maintains the functional profile of tocosh, providing measurable anti-inflammatory activity and supporting its potential characterization as a functional food with demonstrated bioactive properties.

## Figures and Tables

**Table 1:** Determination of the IC_50_ of the selenium-enriched tocosh on human colorectal adenocarcinoma (Caco-2).

IC_50_		
Sample		IC_50_ (mg/mL)
	Tocosh CNT	>200
Se-enriched tocosh	4 h	>200
	16 h	>200
	28 h	22.8

Values are expressed as means ± SD of at least three independent replicates.

## Data Availability

The original contributions presented in the study are included in the article, further inquiries can be directed to the corresponding author.

## Abbreviations

The following abbreviations are used in this manuscript

Se	Selenium
NO	Nitric oxide
YEPD	Yeast extract peptone dextrose
GPx	Glutathione peroxidases
ROS	Reactive oxygen species
CFUs	Colony-forming units
ICP-OES	Inductively coupled plasma optical emission spectrometry
RSD	Standard deviations
eGI	Estimated glycemic index
PBS	Phosphate-buffered saline
UV–Vis	UV–visible spectrum
AUC	Area under the curve
SGID	In vitro simulated gastrointestinal digestion
DPPH	Radical scavenging activity
